# Dairy product purchasing in households with and without children

**DOI:** 10.3168/jdsc.2020-19305

**Published:** 2021-01-15

**Authors:** Mario Ortez, Courtney Bir, Nicole Olynk Widmar, Jonathan Townsend

**Affiliations:** 1 Department of Agricultural Economics, Purdue University, 403 W. State St., West Lafayette, IN 47907; 2 Department of Agricultural Economics, Oklahoma State University, 529 Ag Hall, Stillwater 74078; 3 Department of Veterinary Clinical Sciences, Purdue University, 625 Harrison Street, West Lafayette, IN 47907

## Abstract

Dairy products are considered a staple in the American diet due in part to their nutritious content and appealing flavor. Consumption patterns may differ among age segments influenced by preferences for fat content and taste in dairy. Furthermore, having children in their household may influence the amount, frequency, and type of dairy products that a consumer chooses to purchase. This study finds that households that frequently bought food for children generally purchased larger quantities of fluid milk and more fluid milk with a higher fat content. Households with children also bought yogurt more frequently than other households. Labeling information of interest to the largest proportion of respondents was price; unsurprisingly, expiration date was second most common on meat, eggs, or milk product packaging. Although the scientific and cultural debate of the benefit or harm of consuming dairy continues, it is clear that the segment of the population that frequently purchases food for children is interested in dairy as part of the diet of the children. It would be interesting to see whether this finding has a spillover effect on the consumption habits of adults making the purchases. Future studies can build on this work by evaluating whether there is a spillover effect from purchasing specifically for children and the general dairy and protein product purchasing habits of those households.

## Highlights

• Households buying food for children bought dairy differently
• Households buying food for children bought larger quantities of fluid milk
• Households buying food for children bought fluid milk with higher fat content
• Households frequently buying food for children bought yogurt more often
• Price, expiration date, and nutrition were the most-read meat/milk/egg label info

## Graphical Abstract

**Figure:**
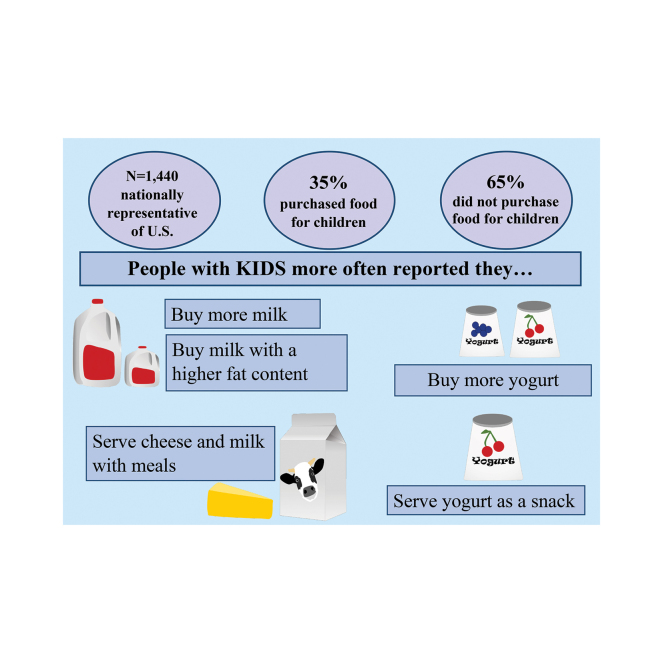
**Summary:** Households that frequently purchased food for children had different dairy buying behavior compared with other households. Households with children reported buying larger quantities of fluid milk and yogurt, and their chosen fluid milk had a higher fat content. Meat, egg, and milk product package labeling information of interest to the highest percentage of households was price, followed by expiration date and nutritional information.

Dairy consumers in the United States are exposed to and interact with information from different government guidelines, medical associations, and popular news and social media. That information, combined with their cultural background and food preferences can influence their buying behavior. In spite of the overall decline in fluid milk consumption in the United States, there is still an overall perception that dairy milk is good for children (Bryant, 2018). Therefore, even if a consumer does not prefer dairy themselves, they might still choose to purchase dairy products for children in their households. The difference in perception of “goodness” of dairy for different age segments of the population raises the question of whether households buying food for children have different buying patterns than households that do not buy food for children. Private market reports have indirectly hinted at this question, finding that 94% of millennial parents purchased dairy milk in a 3-mo period in 2018 (Bryant, 2018). Additionally, the Mintel Analysis (Bryant, 2018) reported that from 2013 to 2018, the average number of glasses of milk consumed was higher in households with at least one child under the age of 18 by at least one glass per day.

Dairy products contribute nutrients to the diet, including calcium, phosphorus, vitamin A, vitamin D (in products fortified with vitamin D), riboflavin, vitamin B_12_, protein, potassium, zinc, choline, magnesium, and selenium (Dietary Guidelines for Americans; USDA-DHSS, 2015–2020). Furthermore, evidence suggests that intake of milk and milk products is linked to improved bone health, especially in children and adolescents, and to a reduced risk of cardiovascular disease and type 2 diabetes and lower blood pressure in adults (Dietary Guidelines for Americans; USDA-DHSS, 2010). Dairy products, including milk, yogurt, and cheese, are and traditionally have been listed as key recommendations for a healthy eating pattern (Dietary Guidelines for Americans; USDA-DHSS, 2000, 2010, 2015–2020, Phillips and Briggs, 1975). Despite the health benefits and intake recommendations, current consumption levels of dairy in the United States are below recommendations for all age groups, except for the majority of young children, 1 to 3 yr old (Dietary Guidelines for Americans; USDA-DHSS, 2015–2020).

Although dairy consumption levels are below prominent government organization recommendations, dairy milk and dairy milk products, including fluid milk, yogurt, cheese, and butter, are familiar to the American diet (Bryant, 2018). Although all dairy product consumption per capita in the United States has trended upward since 1975, consumption of fluid milk per capita has been declining while that of other dairy products has largely increased (Widmar, 2020). Replacement, questioning of traditionally accepted attributes, and digestibility are some of the common reasons for the decline in fluid dairy consumption that surface in popular media (Ferdman, 2014; The Guardian, 2020). The decline in fluid milk consumption suggests that fluid milk drinking is complicated beyond just nutrition facts and is likely the result of tastes, preferences, past experiences, competition with other beverage options, and a multitude of other factors.

Children may also have a say in what they eat, and some may even have a strategy to get what they want. The influence of children on parents' buying behavior has been analyzed. Noble et al. (2007) explored discrepancies between the nutritional knowledge of Australian parents of preschool children and their actual food purchase and preparation behavior and found that “treats” or “bribes” were the main motivational force behind the purchase of unhealthy foods. In contrast, the decision to purchase healthy foods was motivated by “good parenting.” In Noble et al. (2007), parents indicated that they were aware of the implications of providing their children with healthy and unhealthy foods, which illustrates that scientific facts are not the lone drivers of decision making when purchasing food.

The objective of this study was to determine the dairy buying behavior of US households and further determine whether households that frequently purchased food for children had a different dairy buying behavior than other households. The hypothesis is that households that frequently buy food for children have a different dairy buying pattern compared with households that do not frequently buy food for children. This study also compared different pieces of information on food labels assessed by buyers in households that frequently purchase food for children compared with that in households that do not frequently purchase food for children.

The survey instrument was administered from June 11 to June 21, 2019, using Qualtrics (https://www.qualtrics.com/), an online survey tool, to collect household demographic information and dairy product purchasing behavior from US residents. Kantar (https://www.kantar.com/), a London-based company that hosts an online panel database, was used to obtain survey respondents through the use of their large opt-in panel database. Respondents were required to be 18 yr of age or older to participate. The sample was targeted to be representative of the US population in terms of sex, age, income, education, and geographical region of residence as defined by the US Census Bureau (2016). Regions of residence were defined as in the Census Bureau Regions and Divisions.

In total, 1,440 completed surveys were collected; 511 respondents indicated they frequently purchased food specifically for children, whereas 929 indicated they did not. Of the 1,440 respondents, 521 indicated that they had at least one child in the household, 912 indicated they did not have children in their household, and 7 respondents gave null answers. Seventy-eight households indicated they had children in their household but did not buy food specifically for them. This discrepancy between households that had children in the household but indicated not buying food for children may be explained by siblings, nephews, nieces, or grandchildren who may be living in the same house as the respondent but the respondent is not the adult in charge of buying food. It could also be that those respondents are implying that they do not buy food “specifically” for children; in other words, children eat the same products as the rest of the household. All respondents were asked questions regarding their milk and yogurt purchasing behaviors and habits. Respondents who indicated they frequently purchased food for children were asked further questions regarding their yogurt, fluid milk, cheese, and ice cream buying habits. Frequencies were calculated for categorical variables and means were calculated for continuous variables. The test of proportions (Acock, 2018) was conducted to determine the statistical representativeness of the sample compared with the US Census, as well as to test for differences between households with versus without children and those buying food for versus not buying food for children.

The sample of respondents, summarized in Table 1, closely matched the US population, although the following categories in the sample were statistically lower than in the US Census: males, 18–24 years old, $100,000 and higher income, did not graduate from high school, attended college, no degree earned, and residences in the Midwest. The following categories were statistically higher than the US Census: 35–44 years old, graduated from high school, did not attend college, and attended college, associate or bachelor's degree earned. The overall mean number of adults and children per household was 2 adults and 0.6 children. Respondents who indicated they do not frequently buy for children had a mean of 1.9 adults and 0.1 children per household. Respondents who indicated they frequently buy for children had mean of 2.2 adults and 1.5 children per household.

**Table 1:** Sample demographics (n = 1,440)

Demographic variable	% of all respondents (n = 1,440)	US Census (% of population)	Respondents (%):	
			Purchasing food for children (n = 511)	Not purchasing food for children (n = 929)
Sex				
Male	46*	49	48	44
Age (yr)				
18–24	9*	13	10	8
25–34	19	18	37	10
35–44	18*	16	31	12
45–54	16	17	13	18
55–65	17	17	6	23
>65	20	19	3	29
Household income				
$0–$24,999	23	22	14	28
$25,000–$49,999	24	23	19	28
$50,000–$74,999	18	17	20	17
$75,000–$99,999	13	12	19	9
$100,000 and higher	22*	26	28	19
Education				
Did not graduate from high school	5*	13	7	5
Graduated from high school, did not attend college	32*	28	28	34
Attended college, no degree earned	18*	21	16	20
Attended college, associate's or bachelor's degree earned	31*	27	32	31
Attended college, graduate or professional degree earned	13	12	17	11
Mean (SD) number of adults	2.0 (1.8)		2.2 (2.0)	1.9 (1.6)
Mean (SD) number of children	0.6 (1.3)		1.5 (1.5)	0.1 (0.8)
Region				
Northeast	18	18	20	18
South	37	38	41	38
Midwest	21	21	17	23
West	22	24	23	22

* Statistically different, at the 0.05 level, from the percentage of the US population (US Census Bureau, 2016).

Out of the 511 respondents that reported they frequently purchase food for children, 58% reported that price is the piece of information they assess in reviewing meat, egg, or milk products. Fifty-four percent reported product expiration, 44% reported nutritional information, 33% reported food safety information, 26% reported local food labeling, 22% reported animal welfare information, 6% reported none, and 3% reported other. Out of the 929 respondents that reported they do not frequently purchase food for children, 71% reported that price is the piece of information they assess in reviewing meat, egg, or milk products, 65% reported product expiration, 36% reported nutritional information, 24% reported food safety information, 20% reported local food labeling, 13% reported animal welfare information, 15% reported none, and 2% reported other. The differences in responses between households purchasing for children and other households were statistically different for each category except for “other.”

Households that frequently purchase food for children generally bought more fluid milk than other households. Sixteen percent of households who did not frequently purchase food for children also did not buy fluid dairy milk compared with 4% of households that frequently purchase food for children. Figure 1 illustrates in 2 graphs—one for households purchasing for children (top) and one for households not frequently purchasing for children (bottom)—the quantity of fluid milk purchased and fat content that respondents indicated they bought on a per-week basis. On average, households that frequently bought food for children bought more fluid milk, and that milk had a higher fat content than households that did not frequently buy food for children. Respondents who indicated that they purchased whole milk also purchased a larger quantity, which can be seen in households that frequently purchase food for children, and to a lesser extent in households that did not frequently purchase food for children. For example, out of all respondents who indicated they buy food for children and buy whole milk, 38% of them indicated they buy more than 2 gallons per week, compared with 19%, 26%, and 30% for fat-free skim milk, 1% low-fat, and 2% reduced-fat respectively.

**Figure 1:**
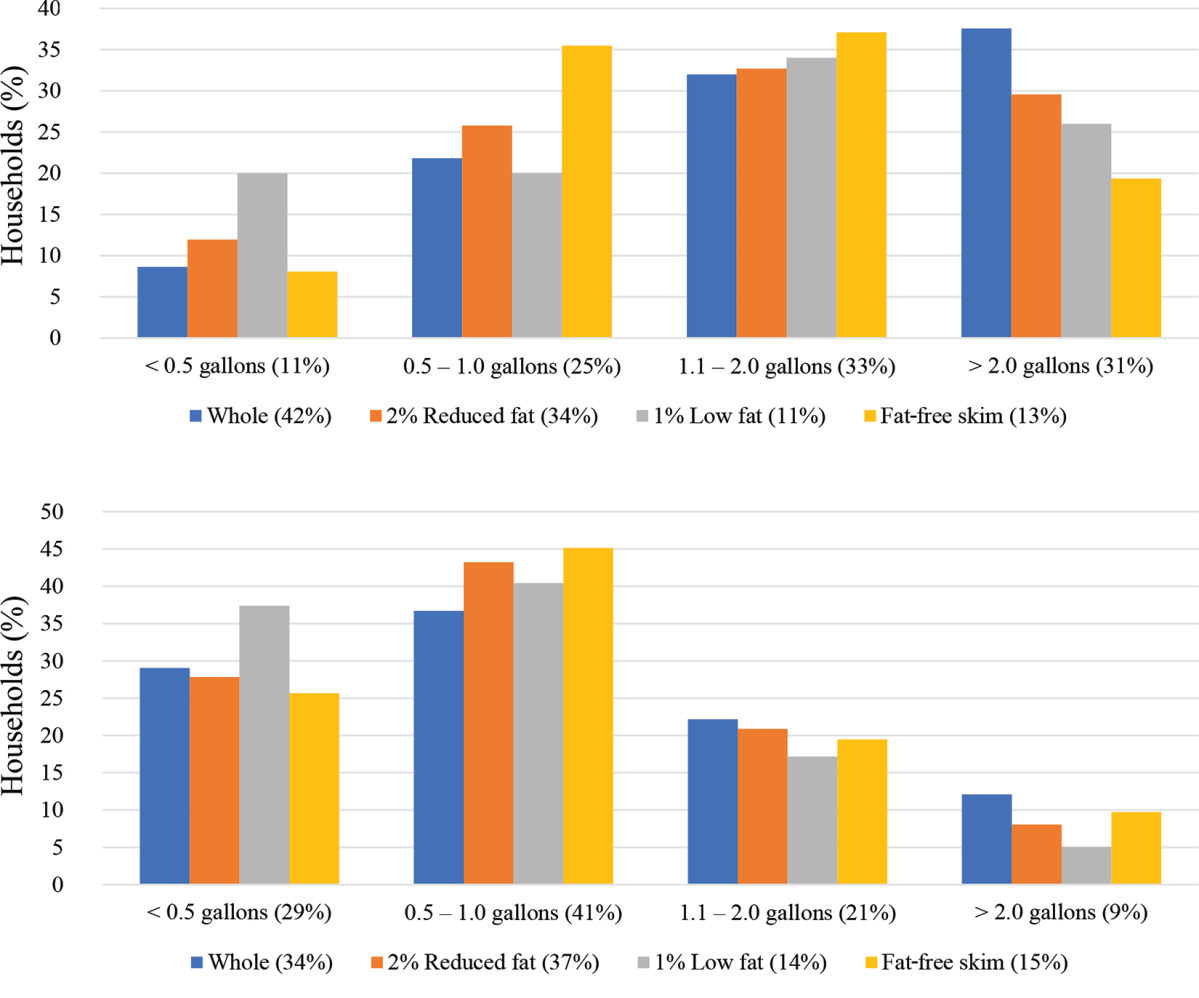
(Top) Percentage of households making weekly purchase quantities by fat content category (buying for children, n = 468); (bottom) percentage of households making weekly purchase quantities by fat content category (not buying for children, n = 733).

The Dietary Guidelines for Americans (USDA-DHSS, 2015–2020) recommend drinking reduced-fat milk, whereas the American Academy of Pediatrics (AAP) advises that children between 12 and 24 mo should drink whole milk (Muth, 2019). A distinction for children between 12 and 24 mo was not made in the 2015–2020 Dietary Guidelines. The consensus seems to be that fat-free and low-fat dairy products provide the same nutrients but less fat; thus, the recommendations for dairy intake come in the form of fat-free or low-fat products for children and adults (Dietary Guidelines for Americans; USDA-DHSS, 2000, 2010, 2015–2020). Nevertheless, consumption of regular-fat milk and cheese was more prominent among Americans according to the 2010 Dietary Guidelines compared with lower-fat alternatives. Furthermore, fluid milk sales seem to have experienced a shift in trends after 2011, when whole fluid milk sales started trending higher, and fluid milk sales of 2%, 1%, and skim milk started trending lower (Widmar, 2020). The AAP has changed its recommendation over time. In 2008, revising a 1998 recommendation, they recommended that children between 12 and 24 mo for whom being overweight or obese is a concern or who have a family history of obesity should consume reduced-fat milk (Daniels and Greer, 2008). In the popular media, the case for whole milk in infants goes beyond the fat and nutrition content to its taste. Many argue that full-fat milk tastes better, hence it is easier for children to get used to it, which in turn helps them obtain the calories and fat they need for growth and brain development (Iannelli, 2020). This illustrates the potential confusion of different segments of the population in the United States in terms of how dairy fits in their healthy diets and that of their children. This study sought to reconcile the different pieces of information and historical trends in dairy consumption with the actuality of buying behavior of households that frequently purchase food for children and households who do not.

Several studies have delved deeper into the merits of regular-fat milk compared with lower-fat alternatives. Vanderhout et al. (2020) conducted a meta-analysis to evaluate the relationship between cow-milk fat consumption and risk of obesity in children between 1 and 18 yr around the world. Using observational evidence, their results suggest that intake of higher-fat cow milk is associated with lower odds of children being overweight; hence, guidelines that recommend reduced-fat milk for children might not lower the risk of childhood or adolescence obesity. Other studies have examined the idea that the consumption of dairy products has a decreasing effect on weight in children and adolescents (Barba et al., 2005; Moore et al., 2008; Abreu et al., 2014), which illustrates that the debate on the merits of dairy consumption in children is not yet settled.

Frequency of yogurt purchases varied depending on the yogurt category and whether or not households purchased food for children. The share of households that frequently purchase food for children and bought yogurt weekly was larger than the share of households who reported they do not frequently purchase food for children and bought yogurt weekly (Table 2). For traditional yogurt, the largest share of households that frequently purchased food for children mostly bought it weekly. Households that do not frequently purchase for children were more prominent in the monthly frequency. Traditional yogurt in large tubs was most often bought weekly by households that frequently bought food for children, whereas Greek yogurt in large tubs was bought most frequently in the “never” category for the same group. For households that did not frequently purchase food for children, traditional and Greek yogurt in large tubs was most frequently selected in the “never” category.

**Table 2:** Yogurt purchasing preferences and frequency (percentage of respondents)

Product	Weekly (%)	Monthly (%)	Never (%)	I do not know this product/have never heard of it
Individual traditional yogurt cups (n = 951)	48	34	16	2
Household buys for children (n = 411)	65	26	8	1
Household does not buy for children (n = 540)	35	41	21	3
Individual Greek yogurt cups (n = 951)	29	40	28	3
Household buys for children (n = 411)	37	42	19	2
Household does not buy for children (n = 540)	23	39	35	3
Traditional yogurt in large tubs (n = 951)	23	23	49	5
Household buys for children (n = 411)	41	26	30	3
Household does not buy for children (n = 540)	10	21	63	6
Greek yogurt in large tubs (n = 951)	17	26	50	7
Household buys for children (n = 411)	28	33	33	6
Household does not buy for children (n = 540)	8	20	64	8
Drinkable yogurt (n = 951)	22	18	54	7
Household buys for children (n = 411)	40	27	29	5
Household does not buy for children (n = 540)	8	11	73	8
Yogurt tubes (e.g., Go-Gurt) (n = 951)	18	22	53	7
Household buys for children (n = 411)	35	40	21	4
Household does not buy for children (n = 540)	5	9	77	9
Child-portioned yogurt cups, drinks, tubes, etc. (n = 951)	22	16	55	7
Household buys for children (n = 411)	44	29	25	3
Household does not buy for children (n = 540)	6	6	79	9

Respondents who indicated that they frequently purchased for children (n = 511) were asked questions regarding cheese, yogurt, and milk buying behavior under different product categories (Table 3). The questions specifically addressed whether the dairy food was bought for snacks, as part of breakfast, as part of lunch, as part of dinner, or in recipes (“never” was also an option). The highest percentage of households reported buying the following items “for snacks”: cheese sticks, cubed cheese, cheeseballs, and blocks of cheese; the following item “as part of lunch”: cheese slices; the following items “as part of dinner”: shredded cheese and grated cheese (e.g., Parmesan); and finally, the following items under the “never” category: cheese wheels, liquid cheese products (cheese dips), and spray (canned cheese). The highest share of households reported buying all categories of yogurt for snacks. Other than never, the highest share of households reported buying regular whole milk, regular reduced-fat 2% milk, regular low-fat 1% milk, regular skim milk, flavored reduced-fat 2% milk and single-serving milk as part of breakfast. Flavored whole milk, flavored low-fat 1% milk, and flavored skim milk were most often purchased for snacks. One consideration that should be highlighted for future work is the possible intersection of household purchases for children, especially dairy and fluid milk, and school lunch program participation. The age of the child, type of school program (whole- or half-day school programs for younger children, for example), and geographic region may all affect the school lunch programs available. However, the possibility of dairy consumption/purchasing in households with children being affected by school lunch consumption or participation in reduced-cost school lunch is deserving of attention, especially in light of today's political and economic climate surrounding such programs.

**Table 3:** Dairy products purchasing for children (% of respondents)

Product	For snacks	As part of breakfast	As part of lunch	As part of dinner	In recipes	Never
Cheese (n = 457)						
Cheese sticks	49	15	16	8	3	9
Cubed cheese	40	16	11	7	8	19
Shredded cheese	18	15	15	25	23	4
Cheese wheels	23	17	12	8	6	35
Cheese balls	32	14	13	6	5	30
Blocks of cheese	31	15	13	15	14	11
Cheese slices	21	17	39	11	7	6
Liquid cheese products or cheese dips	26	16	14	9	8	28
Spray or canned cheese	32	15	12	5	4	32
Grated cheese (e.g., Parmesan)	16	17	12	30	19	7
Yogurt (n = 420)						
Traditional yogurt	36	23	17	9	5	10
Greek yogurt	28	22	15	7	5	22
Drinkable yogurt	34	15	16	7	5	23
Yogurt tubes	40	17	15	8	3	17
Child-portioned yogurt cups	36	20	20	5	4	16
Child-portioned drinkable yogurt	34	17	15	7	4	23
Child-portioned yogurt tubes	36	18	17	7	4	18
Fluid milk (n = 447)						
Regular whole milk	18	34	13	10	7	17
Regular reduced-fat (2%) milk	20	26	15	9	6	24
Regular low-fat (1%) milk	17	20	13	9	5	35
Regular skim milk	18	20	11	8	6	37
Flavored whole milk	23	22	15	8	4	28
Flavored reduced-fat (2%) milk	22	23	11	10	5	29
Flavored low-fat (1%) milk	18	17	14	7	5	38
Flavored skim milk	21	15	13	8	4	40
Single-serving milk	21	23	13	8	4	30

A few implications regarding cheese, yogurt, and milk buying behavior from households that frequently bought food for children were that cheese and milk are bought most frequently for part of a meal and yogurt is bought most popularly for a snack. Additionally, a greater consistency in purchasing habit was observed for milk (which was most often purchased for breakfast and snacks) and yogurt (which was most often purchased as a snack) compared with cheese. Cheese is purchased for more diverse reasons, including snacks, breakfast, lunch, dinner, or never. This study found differences in the dairy buying behavior of households who frequently purchase food for children compared with other households. Households that frequently purchase food for children generally bought more fluid milk and more fluid milk with a higher fat content; they also bought yogurt more frequently. Regarding egg, milk, and meat labeling, this study found that the largest share of households reported reviewing price, expiration date, and nutritional information, in that order. Practitioners in the dairy industry as well as policy makers can take from these results that their efforts to influence consumption of dairy should include tailoring them to the specific household segments being targeted. The findings regarding the most read information on meat, milk, and eggs labels can help inform product labeling.
